# Construction of exogenous methanol, formate, and betaine modules for methyl donor supply in methionine biosynthesis

**DOI:** 10.3389/fbioe.2023.1170491

**Published:** 2023-03-31

**Authors:** Zhen-Yang Shen, Yi-Feng Wang, Li-Juan Wang, Bo Zhang, Zhi-Qiang Liu, Yu-Guo Zheng

**Affiliations:** ^1^ The National and Local Joint Engineering Research Center for Biomanufacturing of Chiral Chemicals, Zhejiang University of Technology, Hangzhou, China; ^2^ Key Laboratory of Bioorganic Synthesis of Zhejiang Province, College of Biotechnology and Bioengineering, Zhejiang University of Technology, Hangzhou, China

**Keywords:** synthetic biology, methionine biosynthesis, multibranched pathway, methyl donor supply, exogenous module, *E. coli*

## Abstract

Methionine is an essential sulfur-containing amino acid that finds widespread applications in agriculture, medicine, and the food industry. However, the complex and multibranched biosynthetic pathway of methionine has posed significant challenges to its efficient fermentation production. In this study, we employed a modularized synthetic biology strategy to improve the weakest branched pathway of methionine biosynthesis. Three exogenous modules were constructed and assembled to provide methyl donors, which are the primary limiting factors in methionine biosynthesis. The first module utilized added methanol, which was converted into 5,10-methylene-tetrahydrofolate for methionine production but was hindered by the toxicity of methanol. To circumvent this issue, a non-toxic formate module was constructed, resulting in a visible improvement in the methionine titer. Finally, an exogenous betaine module was constructed, which could directly deliver methyl to methionine. The final strain produced 2.87 g/L of methionine in a flask, representing a 20% increase over the starting strain. This study presents a novel strategy for improving and balancing other metabolites that are synthesized through complex multibranched pathways.

## Highlights

1. The modularized approach of synthetic biology was implemented to optimize the least efficient branch of the methionine biosynthesis pathway.
2. Three exogenous modules were constructed and subsequently integrated to provide methyl donors for methionine biosynthesis.
3. Methyl donors for methionine biosynthesis were obtained from exogenously added small molecular compounds such as methanol, formate, and betaine.
4. The final engineered strain produced 2.87 g/L of methionine in a flask, exhibiting a 20% increase over the starting strain.

## Introduction

Synthetic biology has been widely applied to construct microbial cell factories, which produce various high-valued chemicals, such as 3-hydroxypropanoic acid (Garg et al., 2023), rhamnolipid (Tiso et al., 2020), and odd-chain fatty acids (Park et al., 2020). Using the modularized strategy, synthetic biology can always solve the problems in traditional metabolic engineering and it brings significant possibilities for microbial transformation (Lee et al., 2015; Choi et al., 2019; Cravens et al., 2019).

Methionine is a crucial sulfur-containing essential amino acid and is used extensively in agriculture, medicine, and the food industry (Bunchasak, 2009; Willke, 2014; Neubauer and Landecker, 2021). Despite its importance, the production of methionine through microbial fermentation is often limited due to the complex and multibranched biosynthetic pathway of methionine. However, microbial fermentation is a promising method for producing methionine, given its use of renewable raw materials and environmental friendliness (Gomes and Kumar, 2005; Willke, 2014; Shim et al., 2017). The titer of methionine obtained through fermentation is generally low, and the process is rarely reported (Gomes and Kumar, 2005; Willke, 2014; Shim et al., 2017). This limitation arises from the complex multilevel regulated and multibranched biosynthetic pathway of methionine (Ferla and Patrick, 2014; Shen et al., 2023).

**FIGURE 1:**
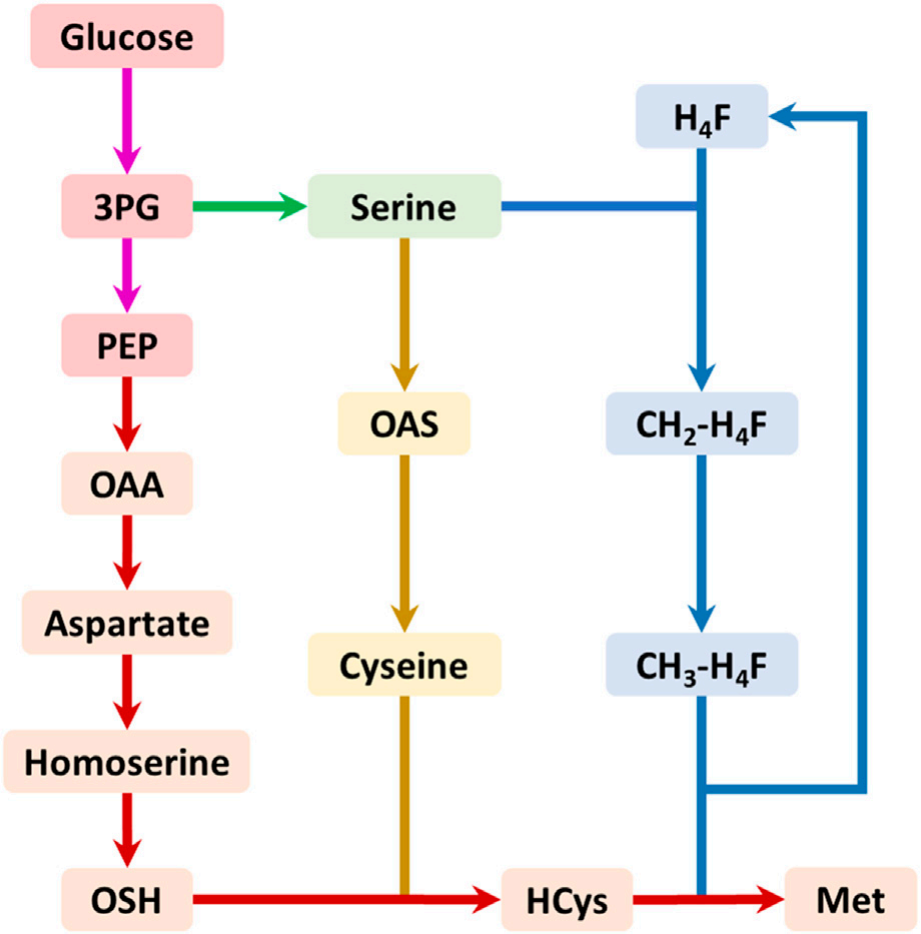
The summary biosynthetic pathway of methionine in *E. coli*. The pink arrows represent the glycolysis pathway; the green arrows represent the serine biosynthesis pathway; the red arrows represent the main pathway of methionine biosynthesis; the yellow arrows represent the cysteine biosynthesis pathway; the blue arrows represent the one-carbon units cycle. Abbreviations: 3PG, 3-phosphate glycerate; PEP, phosphoenolpyruvate; OAA, oxaloacetate; OAS, O-acetyl serine; OSH, O-succinyl homoserine; HCys, homocysteine; Met, methionine; H_4_F, tetrahydrofolate; CH_2_-H_4_F, 5,10-methylene-tetrahydrofolate; CH_3_-H_4_F, 5-methyl-tetrahydrofolate.

The biosynthesis of methionine involves three pathways: the main pathway, the cysteine pathway, and the one-carbon units cycle (Figure 1). The main pathway provides the four-carbon backbone for methionine synthesis, while the cysteine pathway supplies the sulfur and the one-carbon units cycle produces the methyl donor for methionine biosynthesis. The one-carbon units cycle produces the final metabolite 5-methyl-tetrahydrofolate (CH_3_-H_4_F), which serves as the methyl donor for methionine biosynthesis (Figure 1).

Based on the analysis of the biosynthesis pathway, Huang et al. made an effort to reveal and remove the limitations in methionine biosynthesis gradually and constructed a series of engineered *E. coli* for methionine production (Huang et al., 2017; Huang et al., 2018). At first, Huang et al. increased the metabolic flux of the main pathway vastly by knocking out the negative transcription factor (*metJ*) and the competitive pathway of lysine biosynthesis and overexpressing a feedback-resistance mutant of homoserine O-succinyltransferase (MetA), and as a result, the constructed strain attained the preliminary ability of methionine production (9.75 g/L *via* fed-batch fermentation) (Huang et al., 2017). Then Huang et al. improved the two branched pathways by enhancing the availability of precursor (serine) and upregulating the crucial genes in these pathways, such as *cysE* (encoding serine acetyltransferase in cysteine pathway), *metF* (encoding 5,10-methylene-tetrahydrofolate reductase in one-carbon units cycle), and *metH* (encoding methionine synthase in the main pathway), and the final strain was able to produce 16.86 g/L of methionine *via* bioreactor (Huang et al., 2018). On this basis, Shen et al. further subdivided the methionine biosynthetic pathway and revealed more crucial genes as well as the balance between all the branched pathways, in which the abundance of methyl donors (CH_3_-H_4_F) was speculated to be the next bottleneck for methionine production (Shen et al., 2023). However, the CH_3_-H_4_F pathway had the weakest controlling force of metabolic flux (Shen et al., 2023), so the endogenous modification struggled to supply enough methyl donors for methionine biosynthesis. Therefore, the synthetic biology strategy of exogenous module construction, which could utilize added small molecular compounds as raw material to synthesize target metabolite (Zhang et al., 2018; Hu et al., 2022) and hardly influenced the present metabolic flux distribution between these branched pathways, should be considered.

The study used a modularized strategy of synthetic biology to address the issue of lacking methyl donor supply in methionine biosynthesis. Exogenous modules were constructed and assembled in *E. coli IJAHFEBC/pAm (IC/pAm)* as the chassis strain for the supply of methyl donors (Huang et al., 2018). These modules included the methanol module, formate module, and betaine module. The effects of these modules were evaluated by changes in biomass and methionine titer. This approach provides a new strategy for improving and balancing metabolites synthesized through multibranched pathways.

## Materials and methods

### Strains, plasmids, and common techniques

All the strains and plasmids used in this study are shown in Table 1. *E. coli DH5α* was employed for recombinant plasmid construction, and its competent cells were bought from Tsingke Biotechnology Co. Ltd. (Hangzhou, China). *IJAFHEBC* (*IC*) was used as the chassis strain in this work. Plasmid *pAm* and *pACYC184* were employed as the original materials for the plasmid recombination and as the vectors for gene synthesis.

**TABLE 1:** Strains and plasmids used in this study.

	Relevant genotypes	Source
Strain		
*E. coli* DH5α	*F-, φ80dlacZΔM15, Δ(lacZYA-argF) U169, deoR, recA1, endA1, hsdR17(rk-, mk+), phoA, supE44, λ-, thi-1, gyrA96, relA1*	Tsingke^a^
IC (IJAHFEBC)	*E. coli* W3110 derivative, Δ*metI* Δ*metJ* Δ*lysA* Trc-*metH* Trc-*metF* Trc-*cysE* Trc-*serB* Trc*-serC*	Huang et al. (2018)
		
Plasmid		
pACYC184	An expression vector, p15A1 origin of replication, chloramphenicol resistance	Lab collection
pAm	A plasmid derived from pTrc99A carrying *metA* ^fbr^ and *yjeH* along with *serA* ^fbr^	Huang et al. (2018)
184-medh	A plasmid derived from pACYC184 carrying a Ptac promoter along with *medh*	This study
184-faldh	A plasmid derived from pACYC184 carrying a Ptac promoter along with *faldh*	This study
184-fthfl	A plasmid derived from pACYC184 carrying a Ptac promoter along with *fthtl*	This study
184-mthfs	A plasmid derived from pACYC184 carrying a Ptac promoter along with *mthfs*	This study
184-fl-ms	A plasmid derived from pACYC184 carrying a Ptac promoter along with *fthtl* and *mthfs*	This study
184-FMMF	A plasmid derived from pACYC184 carrying a Ptac promoter along with *fthtl*, *mthfs*, *medh,* and *faldh*	This study
184-FMMF-1	A plasmid derived from pACYC184 carrying a Ptac promoter along with *fthtl*, *mthfs*, *medh* ^fbr−1^, and *faldh*	This study
184-FMMF-2	A plasmid derived from pACYC184 carrying a Ptac promoter along with *fthtl*, *mthfs*, *medh* ^fbr−2^, and *faldh*	This study
184-FMMF-3	A plasmid derived from pACYC184 carrying a Ptac promoter along with *fthtl*, *mthfs*, *medh* ^fbr−3^, and *faldh*	This study
pAm-fl-ms	A plasmid derived from pTrc99A carrying *metA* ^fbr^, *yjeH, serA* ^fbr^ along with *fthtl* and *mthfs*	This study
pAm-BsBHMT	A plasmid derived from pTrc99A carrying *metA* ^fbr^, *yjeH, serA* ^fbr^ along with BsBHMT	This study
pAm-TnBHMT	A plasmid derived from pTrc99A carrying *metA* ^fbr^, *yjeH, serA* ^fbr^ along with TnBHMT	This study

^a^ Tsingke, Tsingke Biotechnology Co. Ltd., Hangzhou, China.

All the PCR experiments were implemented using the Phanta Max PCR kit (Vazyme, Nanjing, China). The PCR products were extracted *via* a PCR cleanup kit (Axygen, MA, United States) for further molecular experiments (such as recombinant plasmid construction). The construction of recombinant plasmid was carried out *via* ClonExpress^®^ II One-step Cloning Kit (Vazyme, Nanjing, China).

### Recombinant plasmid construction

Plasmid *184-medh*, *184-faldh*, *184-fthfl*, *184-mthfs*, *pAm-BsBHMT,* and *pAm-TnBHMT* were synthesized by GenScipt (Nanjing, China). Therein, the *medh*, *faldh*, *fthfl,* and *mthfs genes* were codon-optimized and synthesized along with a Tac promoter on the *pACYC184*, respectively. Genes *BsBHMT* and *TnBHMT* were codon-optimized and synthesized using *pAm* as the vector. The constructions of the plasmids, namely, *184-fl-ms*, *184-FMMF,* and *pAm-fl-ms* were carried out by the One-step Cloning Kit, and the process was shown in Supplementary Figure S2. For constructing *184-fl-ms*, the vector *184-fthfl* was linearized *via* PCR. The *mthfs gene* was cloned *via* PCR as well, which used the plasmid *184-mthfs* as the template. The recombinant plasmid *184-fl-ms* was then employed as the vector to construct *184-FMMF* and was linearized by PCR. The *medh* and *faldh* genes were cloned by PCR respectively, which used the corresponding synthesized plasmid as the template (*184-medh* or *184-faldh*). For the construction of *pAm-fl-ms*, the vector *pAm* was linearized by PCR, and these two target genes were cloned from the plasmid *184-fl-ms* by PCR as well. Suitable RBS was chosen for these exogenous genes. The sequences of all synthesized genes, promoters, and RBS used in this work are shown in the Supplementary Material. All the primers for recombinant plasmid construction are listed in Supplementary Table S1.

PCR-based site-directed mutagenesis was implemented to construct *184-FMMF-1*, *184-FMMF-2,* and *184-FMMF-3*. For the construction of *184-FMMF-1*, *184-FMMF* was as the template, and two-round site-directed mutagenesis PCR was carried out by two pairs of primers (Medh A26V F/R and Medh A169V F/R). Then *184-FMMF-2* was constructed *via* the PCR-based site-directed mutagenesis using the *184-FMMF-1* as the template and Medh A31V F/R as primers. For constructing *184-FMMF-3*, the plasmid *184-FMMF-2* was employed as the template for site-directed mutagenesis PCR, in which Medh A368R F/R was used as the primers. The sequences of these primers are shown in Supplementary Table S1.

### Culture condition

LB medium (5 g/L yeast extract, 10 g/L NaCl, and 10 g/L tryptone) was employed for the general culture of *E. coli* (including seed culture). MF medium (2 g/L yeast extract, 20 g/L glucose, 2 g/L Na_2_S_2_O_3_, 1 g/L KH_2_PO_3_, 16 g/L (NH_4_)_2_SO_4_, 1 mL/L salty solution, 0.01 g/L L-lysine, 0.2 mg/L Vb_12,_ and 10 g/L CaCO_3_) was used to determine the methionine titer for the flask test. Therein, L-lysine, Vb_12,_ and CaCO_3_ were sterilized individually. The salty solution contained 5 g/L FeSO_4_·7H_2_O, 500 g/L MgSO_4_·7H_2_O, 5 g/L ZnSO_4,_ and 5 g/L MnSO_4_·8H_2_O. Isopropyl β-D-1-thiogalactopyranoside (IPTG) was used to induce the regulated gene expression for methionine production at the additive amount of 100 µM. When it was necessary, kanamycin (50 mg/L), chloramphenicol (25 mg/L), and ampicillin (100 mg/L) were applied to maintain the stability of plasmids.

For the shake flask fermentation experiment, at first, the single clone of the strain was inoculated into 10 mL LB medium as the seed, and it was incubated at 37°C and 200 rpm for 11 h. Then 1 mL of the seed was added into the large shake flask (0.5 L) that contained 30 mL of MF medium, and it was cultured at 30°C and 150 rpm for 48 h. After fermentation, 1 mL of the broth was collected to determine the biomass (OD_600_) and methionine titer.

### Analytic procedure

The biomass (OD_600_) was measured *via* the spectrophotometer (Ultrospec 3100, Amersham Biosciences, Uppsala, Sweden). The supernatant of fermentation broth was diluted 20 times and filtered for further detection. The titer of methionine was measured *via* an LA8080 amino acid analyzer (Hitachi, Japan).

### RT-qPCR

RT-qPCR was implemented to compare the gene expression level between the plasmid *pAm* and *pACYC184*. *IC/pAm/184-fl-ms* and *IC/pAm-fl-ms* were cultured in 30 mL of MF medium at 150 rpm and 30°C for 18 h. After fermentation, 1 mL of the broth was centrifugated and then frozen using liquid nitrogen. The samples were sent to Tsingke Biotechnology Co. Ltd. (Hangzhou, Zhejiang, China) for RT-qPCR. The 16S rRNA was employed as the internal control. *IC/pAm/184-fl-ms* was the normalization of gene expression.

## Results

### Construction of the exogenous methanol module

The exogenous methanol module was designed to enable the microbial host to use methanol as a carbon source and convert it into methionine. Methanol is an attractive carbon source for microbial fermentation because it is abundant, inexpensive, and can be easily converted into useful products. However, most microorganisms cannot utilize methanol as a carbon source due to the lack of specific metabolic pathways. Therefore, the construction of the exogenous methanol module aimed to introduce the necessary enzymes and genes from other organisms that could enable the microbial host to metabolize methanol and produce methionine.

**FIGURE 2:**
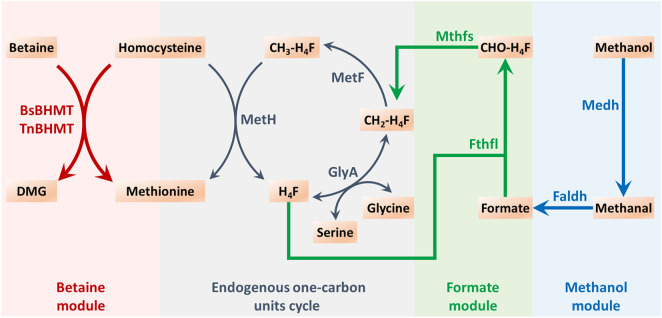
The endogenous one-carbon units cycle and the constructed exogenous modules for methyl donor supply in methionine biosynthesis. Abbreviations: DMG, dimethylglycine; H_4_F, tetrahydrofolate; CH_2_-H_4_F, 5,10-methylene-tetrahydrofolate; CH_3_-H_4_F, 5-methyl-tetrahydrofolate; CHO-H_4_F, formyl-tetrahydrofolate.

According to the study conducted by Yu and Liao (Yu and Liao, 2018), the exogenous methanol module was explored as a means of driving methionine biosynthesis (Figure 2). Four enzymes were utilized in this module, namely, methanol dehydrogenase (Medh, from *Cupriavidus necator N-1*), formaldehyde dehydrogenase (Faldh, from *Methylobacterium. extorquens AM1*), formate tetrahydrofolate ligase (Fthfl, from *M. thermoacetica*), and 5,10-methylene-tetrahydrofolate synthase (Mthfs, from *M. thermoacetica*). These enzymes were encoded by genes that were codon-optimized and synthesized, and the sequences of nucleotides are exhibited in the Supplementary Material. To reduce the burden of *pAm*, a two-plasmid system was employed for methionine biosynthesis. These four exogenous genes were integrated together on the *pACYC184*, which could coexist with *pAm*, resulting in the construction of *IC/pAm/184-FMMF*. An empty vector of *pACYC184* was also introduced into *IC/pAm* for comparison. The result of the shake flask experiment revealed that the initial strain *IC/pAm* produced 2.38 g/L methionine, whereas the two-plasmid system of the empty *pACYC184* did not seem to influence the methionine titer (2.32 g/L), while the introduction of *184-FMMF* reduced it slightly (2.21 g/L).

**FIGURE 3:**
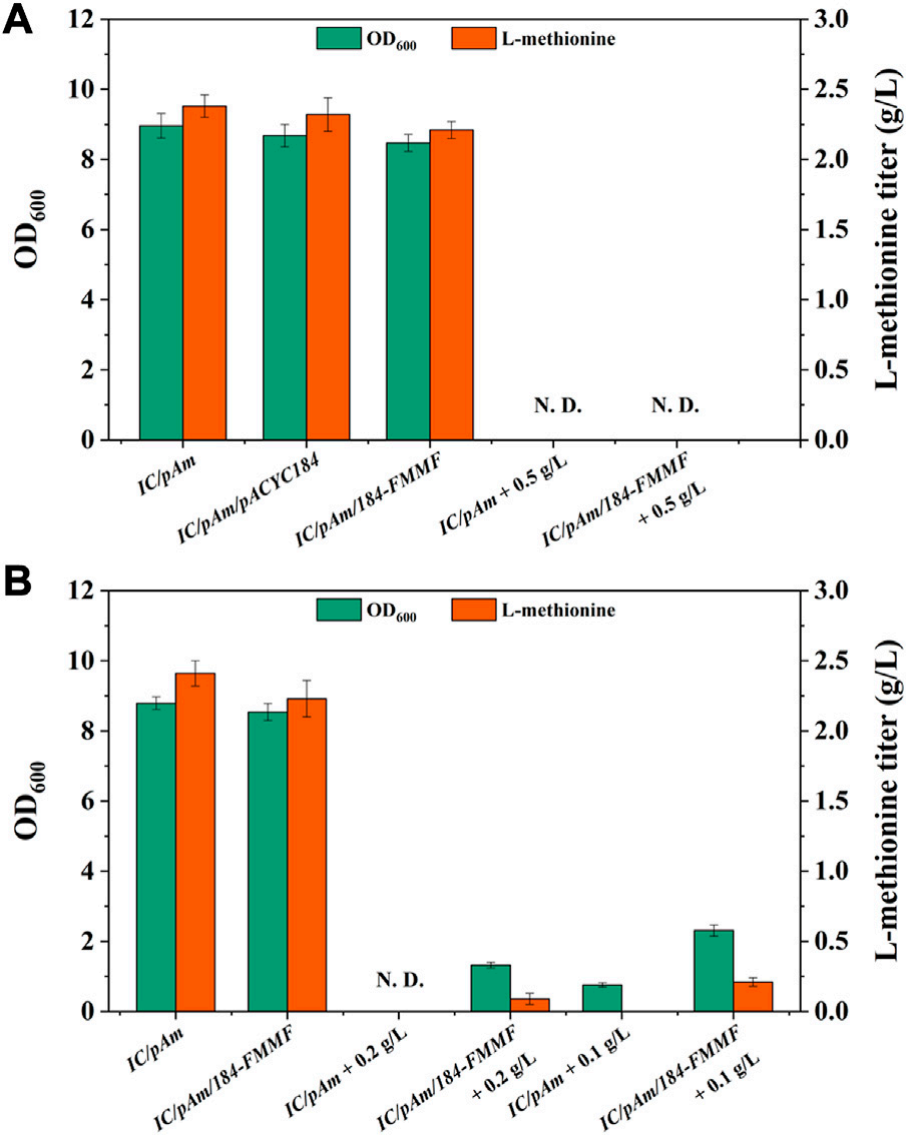
**(A)** The result of methanol module construction. **(B)** The result of optimization of methanol additive amount. Abbreviations: N. D., not detected.

Subsequently, the effect of the exogenous methanol module on methionine production was tested by adding 0.5 g/L of methanol to the medium. However, both the control (*IC/pAm*) and *IC/pAm/184-FMMF* could not grow in the medium containing 0.5 g/L methanol due to the toxicity of methanol, and methionine was not detected in the fermentation broth (Figure 3A). An optimized experiment of methanol concentration was then carried out, and the results indicated that under a methanol concentration of 0.2 g/L, the control strain *IC/pAm* was still unable to grow, while *IC/pAm/184-FMMF* could reach 1.32 of OD_600_ and accumulate 0.09 g/L methionine. When the concentration of methanol had reduced to 0.1 g/L, *IC/pAm* could finally grow to 0.76 of OD_600_, although no methionine was detected in the fermentation broth (Figure 3B). The other strain, *IC/pAm/184-FMMF,* grew better (2.31 of OD_600_) and accumulated more methionine (0.21 g/L) under the 0.1 g/L of methanol concentration (Figure 3B). However, this fermentation behavior was significantly different from that observed in the usual medium, indicating that the added methanol was not converted in a timely manner, and the cell was seriously influenced by the toxicity of methanol.

### Improving the enzymatic activity of medh

**FIGURE 4:**
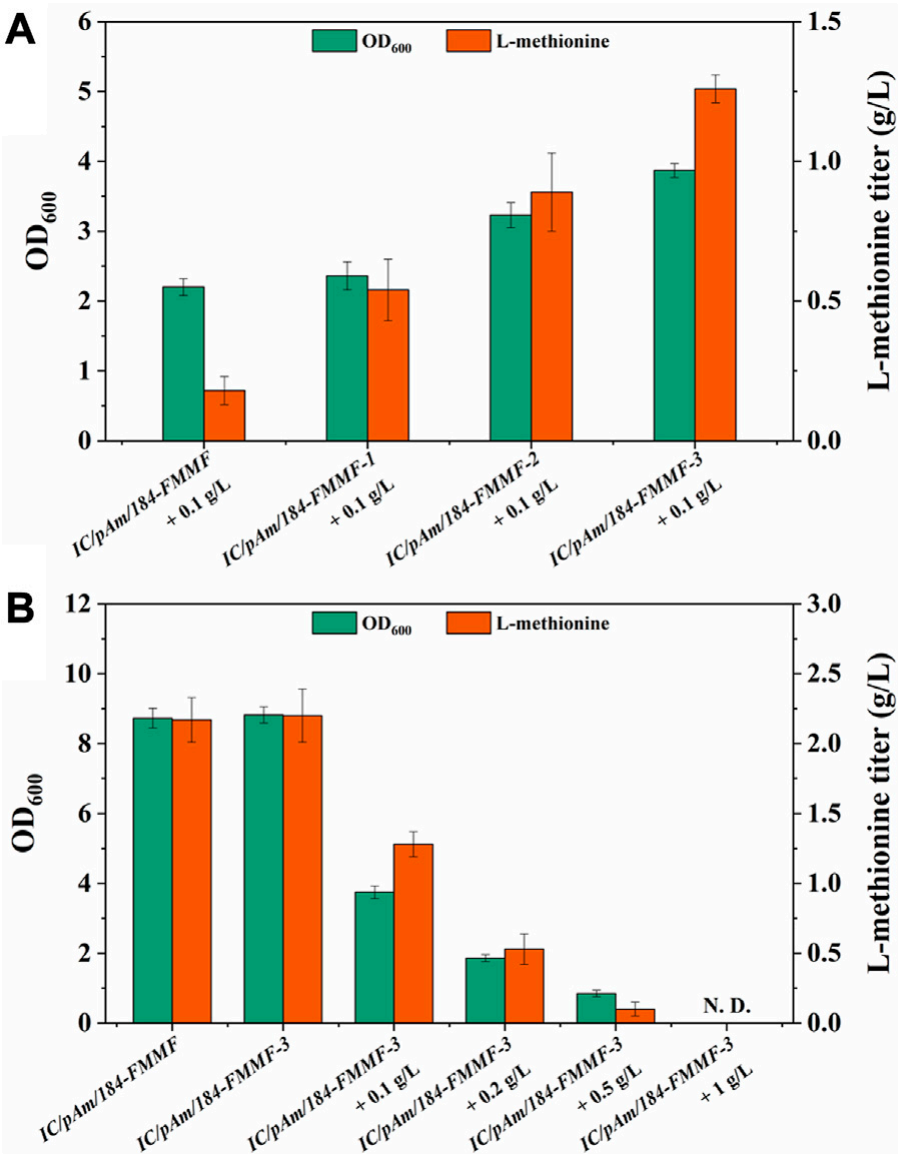
**(A)** The result of Medh modifications. **(B)** The result of optimization of methanol additive amount. Abbreviations: N. D., not detected.

To enhance the conversion efficiency of methanol, modifications were made to methanol dehydrogenase (Medh). Three types of modifications were employed based on previous reports (Yu and Liao, 2018; Zhou et al., 2020) to improve Medh activity. These modifications included A26V/A169V (Medh^fbr−1^), A26V/A31V/A169V (Medh^fbr−2^), and A26V/A31V/A169V/A368R (Medh^fbr−3^), and the corresponding mutant plasmids were named *184-FMMF-1*, *184-FMMF-2*, and *184-FMMF-3*, respectively. The effectiveness of these modifications was evaluated by introducing the mutant plasmids into *IC/pAm* and conducting a fermentation experiment with a methanol concentration of 0.1 g/L. All three mutants showed increased growth and methionine biosynthesis compared to the control (*IC/pAm/184-FMMF*) (Figure 4A), with the highest level of biomass (3.87 of OD_600_) and methionine titer (1.26 g/L) observed in Medh^fbr−3^, which contained four mutant sites. As the number of mutant sites increased, the effectiveness of the modifications became more pronounced.

To further explain the results presented in Figure 4A, it is important to note that methanol is a toxic compound for many microorganisms, including *E. coli.* When methanol is metabolized in cells, it generates formaldehyde, which can damage cellular components such as proteins, lipids, and nucleic acids. To avoid this damage, cells have evolved several mechanisms to detoxify formaldehyde, such as the formaldehyde oxidation pathway, the formaldehyde assimilation pathway, and the formaldehyde resistance pathway. However, these pathways are not always effective, especially at high methanol concentrations.

In the experiment presented in Figure 4B, as the methanol concentration in the medium increased, the toxicity of methanol became more apparent. The growth of *IC/pAm/184-FMMF-3*, even with the Medh^fbr−3^ modification, was hindered by the presence of methanol, and the accumulation of methionine was significantly reduced. At a methanol concentration of 0.5 g/L, the growth of *IC/pAm/184-FMMF-3* was limited to 0.85 of OD_600_ and the methionine titer was only 0.1 g/L, indicating that the presence of methanol was inhibiting the production of methionine. These results suggest that while the Medh^fbr−3^ modification can improve the activity of Medh and enhance the production of methionine under certain conditions, the toxic effects of methanol limit its practical application as a substrate for methionine production in *E. coli*. Further research is needed to identify alternative substrates that can be used for methionine production without compromising the growth and survival of *E. coli*.

### Construction of exogenous formate module

**FIGURE 5:**
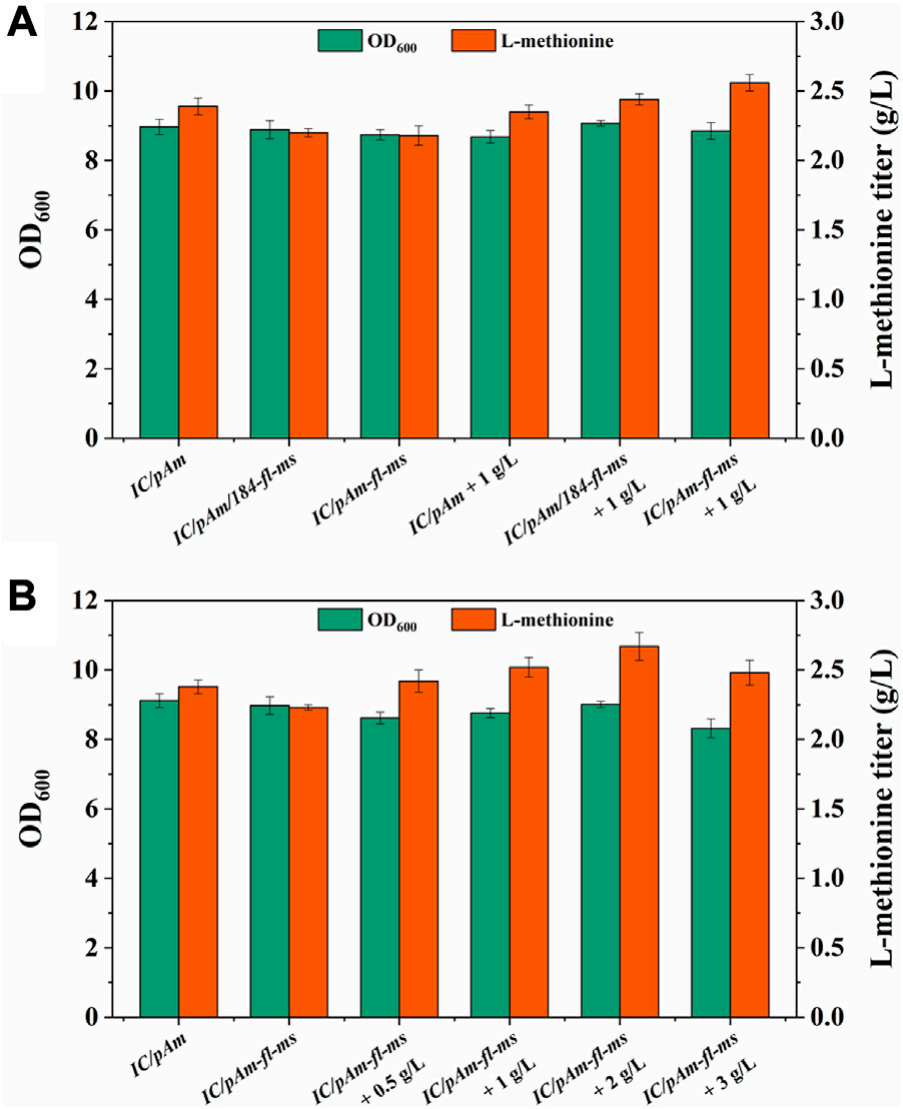
**(A)** The result of formate module construction. **(B)** The result of optimization of formate additive amount.

Methyl donors play a crucial role in cellular metabolism, the exogenous addition of methyl donors may have negative effects on cell growth and metabolism, and thus, a low toxicity methyl donor module was constructed to minimize these effects. Formate, a mesostate of the methanol module was found to be nearly non-poisonous and was selected for this purpose (Figure 2). The two corresponding genes, *fthtl* and *mthfs*, were introduced into the starting strain (*IC/pAm*) *via* a plasmid (*184-fl-ms*), and for a higher expression level, they were also integrated on *pAm* (*pAm-fl-ms*). RT-qPCR experiments confirmed that the transcription levels of these two genes on *pAm* were significantly higher than those on the low-copy-number plasmid *pACYC184* (Supplementary Figure S1). The fermentation behaviors of these constructed strains were analyzed, and overexpression of exogenous genes caused a minor decrease in the methionine titer, regardless of whether it was in a two-plasmid system or a single-plasmid strain (Figure 5A).

To examine the methionine production ability of the exogenous formate module, 1 g/L of ammonium formate was added to the medium. For the control strain (*IC/pAm*), the addition of ammonium formate had almost no effect (Figure 5A). However, the two constructed strains (*IC/pAm/184-fl-ms* and *IC/pAm-fl-ms*) showed a stronger capacity for methionine biosynthesis, with titers of 2.44 g/L and 2.56 g/L, respectively. The strain with a higher expression level of the exogenous genes (*IC/pAm-fl-ms*) exhibited better fermentation behavior than the two-plasmid system strain (*IC/pAm/184-fl-ms*) and was used for an optimization experiment of ammonium formate concentration. As shown in Figure 5B, when the concentration of ammonium formate was below 2 g/L, methionine production increased as the amount of additive increased. The highest titer of methionine was observed at 2 g/L of ammonium formate, reaching 2.67 g/L. However, when the concentration of ammonium formate reached 3 g/L, the methionine titer decreased to 2.48 g/L, suggesting that the conversion efficiency of the module from formate to methionine is relatively low, despite the visible improvement in the methionine titer. These findings provide insight into the construction of low-toxicity methyl donor modules and their potential applications in industrial fermentation.

### Construction of exogenous betaine module

**FIGURE 6:**
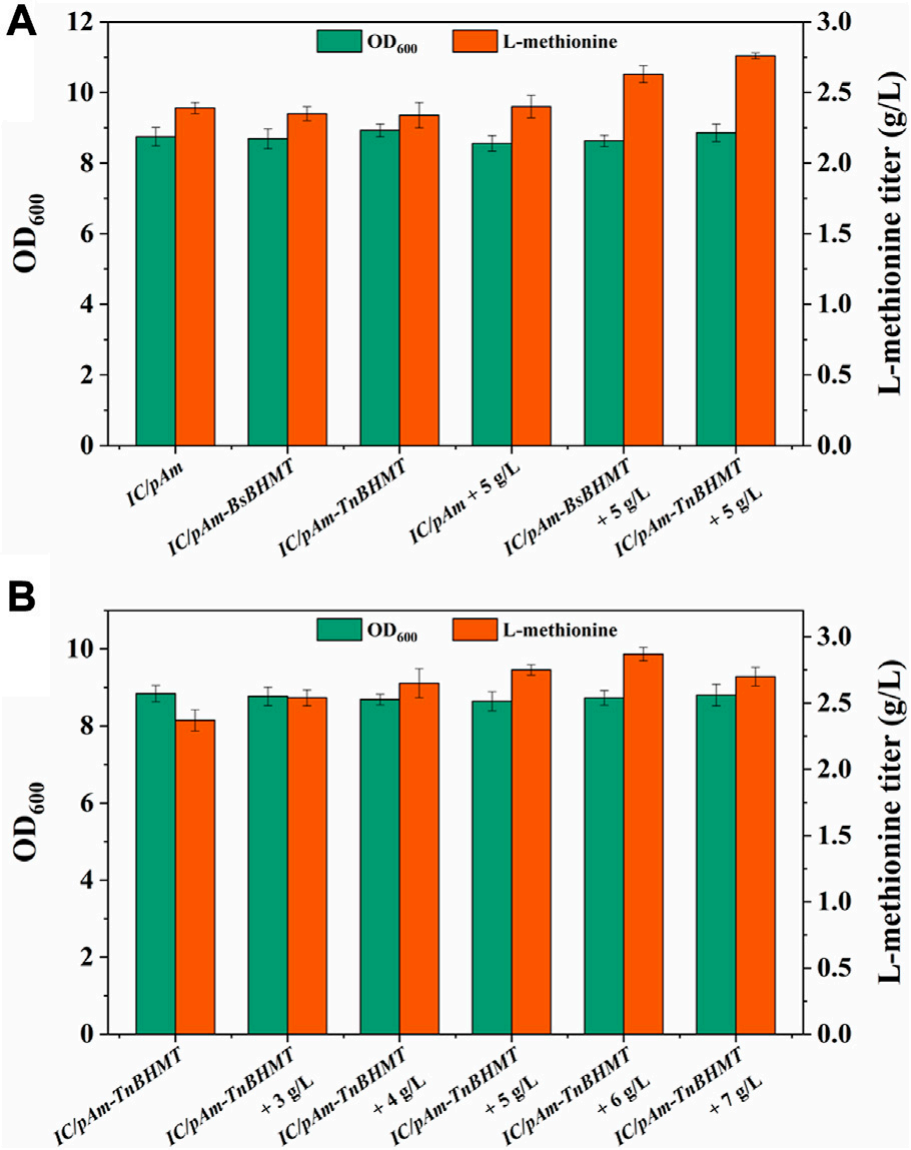
**(A)** The result of betaine module construction. **(B)** The result of optimization of betaine additive amount.

To enhance the supply of methyl donors in a more effective manner, a betaine module was developed based on prior research (Liu et al., 2022), which could directly deliver methyl to homocysteine while producing methionine. This module was powered by betaine-homocysteine methyltransferase (BHMT), which is absent in *E. coli*. Therefore, two exogenous genes encoding different sources of BHMTs were integrated into *pAm*, namely, BsBHMT (from *Bacillus selenitireducens*) and TnBHMT (from *Thioclava nitratireducens*) (Liu et al., 2022). The nucleotide sequences of these genes are provided in the Supplementary Material. The fermentation results are presented in Figure 6A and indicate that overexpression of exogenous genes had a minor impact on the methionine titer (only a 0.05 g/L decrease). However, when 5 g/L of betaine was supplemented, the strains carrying exogenous BHMTs (*IC/pAm-BsBHMT* and *IC/pAm-TnBHMT*) showed significant improvement in the methionine titer (increased to 2.63 g/L and 2.76 g/L, respectively) compared to the control strain (*IC/pAm*), which had negligible changes (Figure 6A).

An optimization experiment on betaine concentration was conducted using *IC/pAm-TnBHMT*, which had better methionine production ability. The results are shown in Figure 6B. In general, the addition of betaine to the medium increased methionine biosynthesis in the strain containing the exogenous betaine module. The maximum methionine titer was achieved at 2.87 g/L when 6 g/L of betaine was supplemented (a 20% increase compared to the initial strain). However, when the betaine concentration was further increased to 7 g/L, the methionine titer decreased to 2.7 g/L. Although the betaine module for the exogenous supply of methyl donors significantly increased the methionine titer, its conversion rate of added betaine was only 6.5%, indicating relatively low efficiency.

## Discussion

The biosynthesis of methionine by microorganisms has always been a challenging task due to the complexity of its regulated and branched biosynthetic pathway (Figure 1). Previous research has identified and overcome various bottlenecks in the pathway, with the primary remaining limitation being the supply of the methyl donors, CH_3_-H_4_F (Huang et al., 2018; Shen et al., 2023). However, due to the intricate branching of the pathway, further modifications pose significant challenges. The endogenous CH_3_-H_4_F pathway is unable to provide sufficient methyl donors for methionine biosynthesis due to weak metabolic flux control (Shen et al., 2023). Therefore, the introduction of an exogenous module that can use small molecular compounds as a source of methyl donors for methionine production is a more feasible approach, as it has minimal impact on the current metabolic flux distribution of the multibranched methionine pathway.

To supply methyl donors for methionine biosynthesis, a methanol module was constructed and introduced to the methionine production strain (*IC/pAm*), which could convert methanol into CH_2_-H4_F_ (Figure 2). The exogenous module was carried by a two-plasmid system, resulting in a slight decrease in the methionine titer due to the overexpression of four new enzymes that consumed amino acids, including methionine, in the cell. High concentrations of methanol were toxic to both the constructed strain and the control, leading to no growth. A gradual reduction in methanol concentration led to better growth and higher methionine production in *IC/pAm/184-FMMF* than the control (*IC/pAm*), indicating the contribution of the exogenous methanol module. However, methanol still had a serious impact on biomass and methionine titer, suggesting low activity of the exogenous enzymes.

To enhance the activity of methanol dehydrogenase, which catalyzes the first step of the exogenous methanol module, three modifications were introduced into Medh. The strain containing the most mutant sites of Medh showed the best fermentation performance and had the highest activity among the mutants, consistent with previous research (Zhou et al., 2020). This mutant strain exhibited superior growth and methionine biosynthesis under low concentrations of methanol compared to the wild type (*IC/pAm/184-FMMF*), demonstrating the importance of enzyme activities in this exogenous module. However, the addition of methanol to the medium still had a significant impact on fermentation behavior, indicating that the activities of exogenous enzymes were insufficient to support methionine production in the presence of methanol toxicity.

To avoid the use of toxic methanol, a formate module was constructed to serve as the second half of the methanol module (Figure 2). To achieve higher expression levels, the genes of the formate module were integrated into the high-copy-number plasmid (*pAm*), as the replication origin of *pACYC184* was a low-copy-number replicon. The results of RT-qPCR and the flask fermentation test confirmed that the sufficient expression of exogenous enzymes was beneficial in improving the conversion efficiency of the exogenous module. After optimization experiments, it was determined that the most suitable concentration of ammonium formate was 2 g/L, which yielded a methionine titer of 2.67 g/L. However, although the methionine production was indeed improved *via* the exogenous formate module, the conversion rate was much lower than the theoretical value. This suggests that in addition to the exogenous methyl donor pathway, there may be limitations in the endogenous one-carbon units conversion pathway (from CH_2_-H_4_F to methionine) that need to be considered.

To supply methyl directly to homocysteine for methionine biosynthesis, an exogenous betaine module was constructed that bypassed the endogenous one-carbon units cycle (Figure 2). Two BHMTs from different sources were employed to carry out this module with TnBHMT (from *T. nitratireducens*), showing higher capability for methionine biosynthesis, which is consistent with previous work (Liu et al., 2022). Optimization experiments showed that the most appropriate amount of added betaine was 6 g/L, resulting in methionine accumulation of 2.87 g/L in the constructed strain, a 20% increase over the starting strain. However, the low conversion rate of added betaine suggested that the activity of the exogenous BHMT was insufficient and required further modification to improve its catalytic ability. In addition, although the conversion efficiency of the two exogenous modules (formate module and betaine module) was low, the strain carrying the betaine module, which delivered methyl directly to homocysteine, produced more methionine with a sufficient exogenous methyl donor supply. This implied that the endogenous one-carbon units conversion pathway (from CH_2_-H_4_F to methionine) also needed improvement.

## Conclusion

In conclusion, the production of methionine by microorganisms is a complex process due to the multibranched biosynthetic pathway and the limited supply of methyl donors. Exogenous modules, such as the methanol, formate, and betaine modules, were constructed to supplement the methyl donor and improve methionine biosynthesis. The optimization experiments showed that the exogenous modules could enhance methionine production to some extent, but the conversion rates were still lower than the theoretical values. The low activities of exogenous enzymes and the limitation of the endogenous one-carbon units conversion pathway were the main factors limiting methionine production.

Future prospects, including further modifications to improve the activities of exogenous enzymes, optimize the expression levels of exogenous modules, and enhance the endogenous one-carbon units conversion pathway, could be employed to redesign the biosynthetic pathway of methionine, which may lead to higher yields and better productivity. Overall, the research in this field holds great potential for the industrial production of methionine, which has broad applications in the food, feed, and pharmaceutical industries.

## Data Availability

The original contributions presented in the study are included in the article/Supplementary Material, further inquiries can be directed to the corresponding author.
