# Supplementary material for: Construction of exogenous methanol, formate, and betaine modules for methyl donor supply in methionine biosynthesis
Source: Front Bioeng Biotechnol. 2023 Mar 31;11:1170491. doi: 10.3389/fbioe.2023.1170491 (PMC10102461; doi:10.3389/fbioe.2023.1170491)
Supplement: Supplementary file 1 [file DataSheet1.docx]

**Table S1. The primers used in this study**

| Primers | Sequence (5’-3’) |
| --- | --- |
| 184-fthfl line F | ATGGAAGCCGGCGGCACCTC |
| 184-fthfl line R | TTTCCTGTGTGAAATTGTTATCCGCTCTTAAAACAGACCGGTAATAAC |
| mthfs F | ATAACAATTTCACACAGGAAACAGACCATGGACCCGGCACAGATTCTG |
| mthfs R | GAGGTGCCGCCGGCTTCCATTTAACGACGTGCGGCTTCCAC |
| 184-fl-ms line F | ATGGAAGCCGGCGGCACCTC |
| 184-fl-ms line R | TTCTTAAAGTTAAACAAAATTATTTCTAGAGTTAACGACGTGCGGCTTCCAC |
| medh+ faldh UF | ATTTTGTTTAACTTTAAGAAGGAGATATACCATGACCCATCTGAATATTGC |
| medh+ faldh UR | AGAGGGAAACCGTTGTGGTCTTACATTGCTGCTGCAAAAATG |
| medh+ faldh DF | GACCACAACGGTTTCCCTCTAC |
| medh+ faldh DR | GAGGTGCCGCCGGCTTCCATTTAGGCTGCGCTGAAGGTTTTATG |
| pAm line F | GGCTGTTTTGGCGGATGAGAG |
| pAm line R | TTAGTACAGCAGACGGGCGC |
| fthfl+mthfs F | GCGCCCGTCTGCTGTACTAAGACCACAACGGTTTCCCTCTAC |
| fthfl+mthfs R | CTCTCATCCGCCAAAACAGCCTTAACGACGTGCGGCTTCCAC |
| Medh A26V F | CCCTGGTTGCGTTCGTGAAACCGGCGCACGCGC |
| Medh A26V R | CGGTTTCACGAACGCAACCAGGGCCAAACAGGG |
| Medh A31V F | TGAAACCGGCGTTCGCGCCCGTAGTCTGGGTGC |
| Medh A31V R | TACGGGCGCGAACGCCGGTTTCACGTGCGCAAC |
| Medh A169V F | TGTTAAGATGGTTATTGTGGATTGGCGCTGTAC |
| Medh A169V R | AATCCACAATAACCATCTTAACATGATTGCTGC |
| Medh A368R F | ACAGAAAGATCGTTGTATGCTGACCAATCCGCG |
| Medh A368R R | TCAGCATACAACGATCTTTCTGTGCATTGCTTG |

**Figure S1. Relative transcription levels of *fthfl* and *mthfs* expressed on *pAm* (compared with *pACYC184*)**





**Figure S2. The process of recombinant plasmid construction**


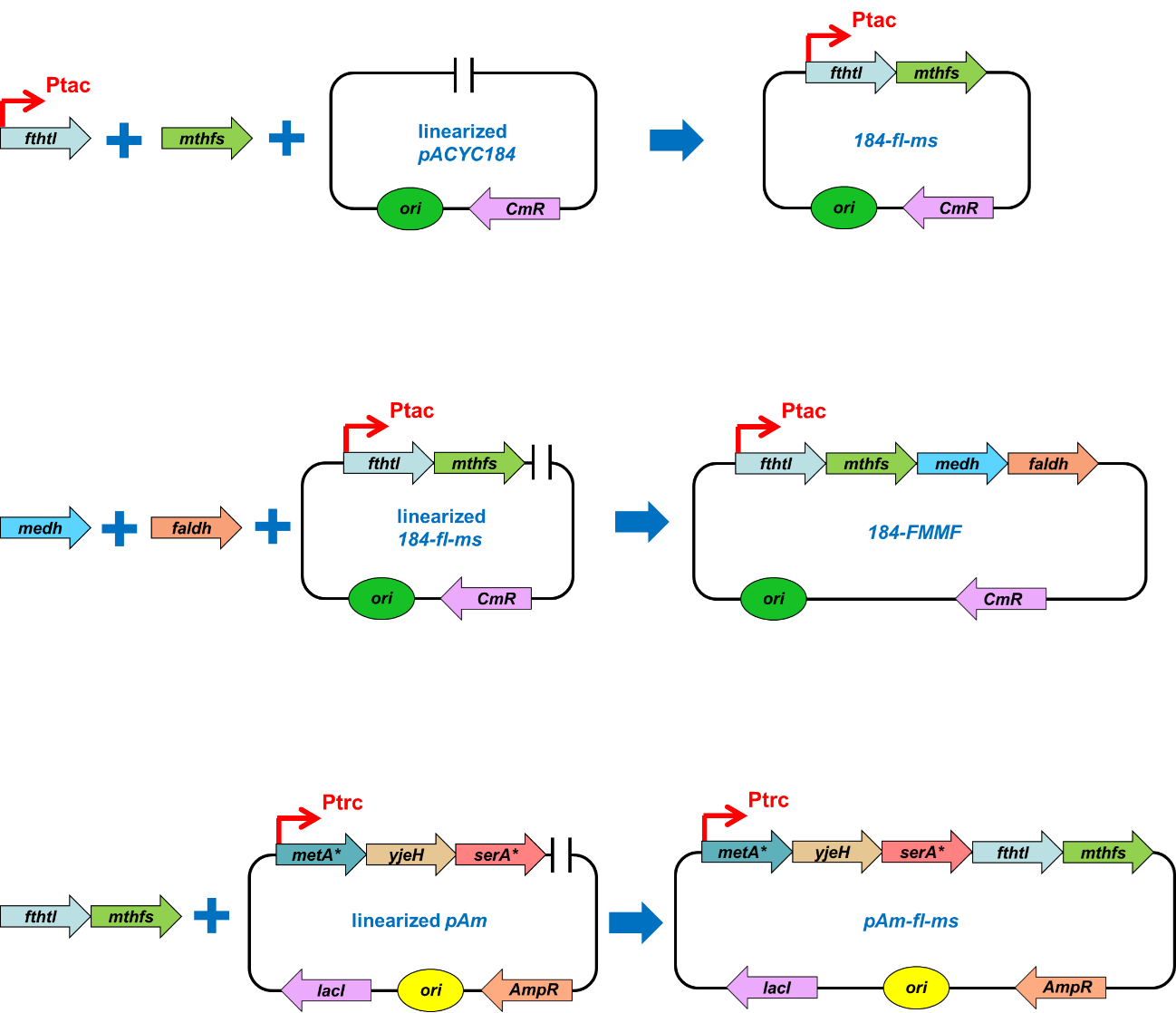


**Sequence S1. The codon-optimized sequence of *medh* (*Cupriavidus necator N-1*) with a promoter Ptac**

CTGGATAACTTGACAATTAATCATCGGCTCGTATAATGTGTGGAGGGAGACCACAACGGTTTCCCTCTACAAATAATTTTGTTTAACTTTCGCGCGCGTAACAGGAGGAATTAA**ATG**ACCCATCTGAATATTGCCAATCGTGTGGATAGCTTTTTCATTCCGTGTGTTACCCTGTTTGGCCCTGGTTGCGCACGTGAAACCGGCGCACGCGCCCGTAGTCTGGGTGCACGTAAAGCCCTGATTGTGACCGATGCAGGCCTGCATAAAATGGGTCTGAGCGAAGTTGTGGCCGGCCATATTCGCGAAGCCGGTCTGCAAGCAGTGATTTTTCCGGGCGCAGAACCGAATCCGACCGATGTTAATGTTCATGATGGCGTGAAACTGTTTGAACGCGAAGAATGCGATTTTATTGTGAGCCTGGGTGGTGGTAGTAGTCATGATTGCGCCAAAGGTATTGGTCTGGTGACCGCCGGTGGTGGCCATATTCGTGATTATGAAGGCATTGATAAAAGCACCGTGCCGATGACCCCGCTGATTAGTATTAATACCACCGCCGGCACCGCAGCCGAAATGACCCGTTTTTGCATTATTACCAATAGCAGCAATCATGTTAAGATGGCAATTGTGGATTGGCGCTGTACCCCGCTGATCGCCATTGATGATCCGAGCCTGATGGTTGCAATGCCGCCGGCACTGACCGCAGCCACCGGTATGGATGCCCTGACCCATGCAATTGAAGCATATGTGAGCACCGCAGCAACCCCGATTACCGATGCATGTGCAGAAAAAGCAATTGTTCTGATTGCAGAATGGCTGCCGAAAGCAGTGGCCAATGGTGACAGTATGGAAGCCCGCGCCGCCATGTGCTATGCACAGTATCTGGCAGGCATGGCATTCAATAATGCCAGCCTGGGCTATGTTCATGCAATGGCACATCAGCTGGGTGGCTTTTATAATCTGCCGCATGGTGTGTGCAATGCAATTCTGCTGCCGCATGTTAGCGAGTTTAATCTGATTGCAGCCCCGGAACGTTATGCACGTATTGCAGAACTGCTGGGTGAAAATATTGGTGGCCTGAGTGCACATGATGCAGCCAAAGCAGCCGTGAGCGCCATTCGTACCCTGAGTACCAGCATTGGCATTCCGGCCGGTCTGGCAGGCCTGGGTGTTAAAGCCGATGATCATGAAGTTATGGCAAGCAATGCACAGAAAGATGCATGTATGCTGACCAATCCGCGTAAAGCAACCCTGGCACAGGTTATGGCCATTTTTGCAGCAGCAATG**TAA**

**Sequence S2. The codon-optimized sequence of *faldh* (*Methylobacterium extorquens AM1*) with a promoter Ptac**

CTGGATAACTTGACAATTAATCATCGGCTCGTATAATGTGTGGAGGGAGACCACAACGGTTTCCCTCTACAAATAATTTTGTTTAACTTTCGCGCGCGTAACAGGAGGAATTAA**ATG**GTTCGTACCGCACATCCGGCCTTTACCGAAAGCGCCAGTGCCCGTTTTCATCGTTGGGCCCCGGCAACCGATGCCAGTAGCAGTGCCCGCCCGCCGGGTAGACGCTCACGTTTATGCATTCATAATAATAATGGCGAACTGCGCATGAGCGGTAATCGCGGCGTGGTTTATCTGGGTAGCGGCAAAGTGGAAGTGCAGAAAATTGATTATCCGAAAATGCAGGACCCTCGTGGTAAAAAGATTGAACATGGCGTGATTCTGAAAGTTGTTAGCACCAATATTTGTGGTAGTGATCAGCATATGGTGCGTGGCCGTACCACCGCCCAGGTGGGTCTGGTGCTGGGTCATGAAATTACCGGTGAAGTTATTGAAAAAGGCCGCGATGTTGAAAATCTGCAAATTGGTGACCTGGTGAGCGTGCCGTTTAATGTTGCCTGTGGCCGCTGTCGTAGCTGCAAAGAAATGCATACCGGTGTGTGCCTGACCGTTAATCCGGCACGTGCCGGCGGCGCATATGGCTATGTGGATATGGGTGACTGGACCGGTGGTCAGGCCGAATATCTGCTGGTGCCGTATGCAGATTTTAATCTGCTGAAACTGCCGGATCGCGATAAAGCAATGGAAAAAATTCGTGATCTGACCTGTCTGAGCGATATTCTGCCGACCGGTTATCATGGTGCAGTGACCGCAGGTGTGGGTCCGGGTAGTACCGTGTATGTGGCCGGTGCAGGTCCGGTTGGCCTGGCAGCTGCCGCAAGTGCCCGCTTACTGGGTGCCGCAGTTGTGATTGTGGGTGACCTGAATCCGGCACGCCTGGCCCATGCCAAAGCACAGGGTTTTGAAATTGCCGATCTGAGTCTGGATACCCCGCTGCATGAACAGATTGCCGCACTGCTGGGTGAACCGGAAGTGGATTGCGCAGTGGATGCAGTTGGCTTTGAAGCACGCGGCCACGGTCATGAAGGCGCAAAACATGAAGCCCCGGCCACCGTGCTGAATAGTCTGATGCAGGTTACCCGTGTGGCCGGTAAAATTGGCATTCCGGGCCTGTATGTTACCGAAGATCCGGGCGCCGTGGATGCAGCAGCCAAAATTGGCAGTCTGAGTATTCGTTTTGGTCTGGGTTGGGCAAAAAGCCATAGCTTTCATACCGGCCAGACCCCGGTTATGAAATATAATCGTGCCCTGATGCAGGCAATTATGTGGGATCGCATTAATATTGCCGAAGTTGTTGGTGTGCAGGTTATTAGCCTGGATGATGCCCCGCGCGGTTATGGCGAATTTGATGCAGGTGTTCCGAAAAAATTTGTTATTGATCCGCATAAAACCTTCAGCGCAGCC**TAA**

**Sequence S3. The codon-optimized sequence of *fthfl* (*Moorella thermoacetica*) with a promoter Ptac**

CTGGATAACTTGACAATTAATCATCGGCTCGTATAATGTGTGGAGGGAGACCACAACGGTTTCCCTCTACAAATAATTTTGTTTAACTTTCGCGCGCGTAACAGGAGGAATTAA**ATG**GAACTGGCCCGCGGTCTGGGTATTCAGGAAGATGAAGTGGAACTGTATGGTAAATATAAAGCAAAAATCAGCCTGGATGTTTATCGTCGCCTGAAAGATAAACCGGATGGCAAACTGATTCTGGTTACCGCAATTACCCCGACCCCGGCAGGTGAAGGCAAAACCACCACCAGCGTGGGCCTGACCGATGCCCTGGCCCGTCTGGGTAAACGCGTGATGGTGTGTCTGCGCGAACCGAGCCTGGGCCCGAGTTTTGGTATTAAGGGTGGCGCAGCCGGTGGCGGTTATGCACAGGTTGTGCCGATGGAAGATATTAATCTGCATTTTACCGGTGACATTCATGCAGTGACCTATGCCCATAATCTGCTGGCAGCAATGGTTGATAATCATCTGCAACAGGGCAATGTTCTGAATATTGATCCGCGCACCATTACCTGGCGTCGTGTGATTGATCTGAATGATCGCGCCCTGCGCAATATTGTTATTGGCCTGGGTGGTAAAGCCAATGGTGTTCCGCGTGAAACCGGCTTTGATATTAGCGTGGCCAGTGAAGTTATGGCATGTCTGTGCCTGGCCAGTGATCTGATGGATCTGAAAGAACGTTTTAGTCGTATTGTTGTGGGTTATACCTATGATGGCAAACCGGTGACCGCCGGTGACCTGGAAGCACAGGGTAGTATGGCCCTGCTGATGAAAGATGCAATTAAGCCGAATCTGGTTCAGACCCTGGAAAATACCCCGGCATTCATTCATGGCGGTCCGTTTGCCAATATTGCCCACGGTTGTAATAGCATTATTGCCACCAAAACCGCACTGAAACTGGCCGATTATGTGGTTACCGAAGCAGGTTTTGGTGCCGATCTGGGTGCAGAAAAATTTTATGATGTGAAATGTCGTTACGCAGGCTTTAAACCGGATGCAACCGTTATTGTGGCCACCGTTCGCGCACTGAAAATGCATGGTGGCGTTCCGAAAAGTGATCTGGCAACCGAAAATCTGGAAGCACTGCGCGAAGGTTTTGCCAATCTGGAAAAACATATTGAAAACATTGGCAAATTCGGTGTTCCGGCCGTTGTGGCAATTAATGCCTTTCCGACCGATACCGAAGCCGAACTGAATCTGCTGTATGAACTGTGCGCAAAAGCAGGTGCAGAAGTTGCCCTGAGTGAAGTTTGGGCAAAAGGCGGTGAAGGCGGTCTGGAACTGGCACGCAAAGTTCTGCAAACCCTGGAAAGTCGTCCGAGCAATTTTCATGTGCTGTATAATCTGGATCTGAGTATTAAGGATAAGATTGCAAAAATCGCAACCGAAATCTATGGCGCCGATGGTGTGAATTATACCGCAGAAGCAGATAAAGCAATTCAGCGCTATGAAAGTCTGGGTTATGGCAATCTGCCGGTTGTGATGGCAAAAACCCAGTATAGCTTTAGTGATGATATGACCAAACTGGGTCGCCCGCGCAATTTTACCATTACCGTTCGCGAAGTGCGTCTGAGTGCAGGCGCCGGTTTTATTGTGCCGATTACCGGTGCAATTATGACCATGCCGGGTCTGCCGAAACGTCCGGCCGCCTGTAATATTGATATTGATGCCGATGGTGTTATTACCGGTCTGTTT**TAA**

**Sequence S4. The codon-optimized sequence of *mthfs* (*Moorella thermoacetica*) with a promoter Ptac**

CTGGATAACTTGACAATTAATCATCGGCTCGTATAATGTGTGGAGGGAGACCACAACGGTTTCCCTCTACAAATAATTTTGTTTAACTTTCGCGCGCGTAACAGGAGGAATTAA**ATG**GACCCGGCACAGATTCTGGATGGTAAAAAGATTGCCGCCGAAGTTCGTGCAGAAGTGAAAGAAGAAGTTAGCCGCCTGAAAGCAGAAGGCATTAATCCGGGTCTGGCCGTGGTGCTGGTTGGTGAAGATCCGGCCAGTCAGGTGTATGTGCGTAATAAGCATCGTGCCTGCGAAGAAGTTGGTATCTATAGTGAAGTTCATCGCCTGCCGGCCGCAACCAGTCAGGCCGAACTGCTGAAACTGATTGATCAGCTGAATAAGGACCCTAAAATTCATGGTATTCTGGTGCAGCTGCCGCTGCCGGATCATATTGATGAAAAGAAAGTGATTGACGCAATTGCCCTGGAAAAAGATGTTGATGGCTTTAGCCCGGCCAATGTTGGTAATCTGGTTATTGGTGACAAATGCTTTTATCCGTGCACCCCGCATGGTTGCATGGTTCTGCTGGAAAAAGCCGGTATTGATCCGAAAGGCAAAAAAGCCGTGGTGGTTGGCCGCAGTAATATTGTGGGCAAACCGGTTGCCATGATGCTGCTGGCCCGCCATGCCACCGTTACCATTTGCCATAGCCGCACCCGCGATCTGGCAGCAGAATGTCGTCAGGCCGATATTCTGATTGCCGCAGTTGGTAAACCGGAACTGATTACCGGCGATATGATTAAGGAAGGTGCCGTTGTTATTGATGTGGGCATTAATCGCGTTGGTGAAAAGAAACTGGTGGGTGACGTTCATTTTGAAAGCGCAGCCCAGAAAGCCGGTTGGATTACCCCGGTGCCGGGCGGTGTGGGCCCTATGACAATTGCCATGCTGCTGAAAAATACCGTGGAAGCCGCACGTCGT**TAA**

**Sequence S5. The codon-optimized sequence of *BsBHMT* (*Bacillus selenitireducens*) with an RBS of Ptac**

GACCACAACGGTTTCCCTCTACAAATAATTTTGTTTAACTTTCGCGCGCGTAACAGGAGGAATTAA**ATG**GCAACCAGCAAACGTACGCTGGAAGAACGTCTGCAGGCCGGTACCGTGATCTGTGCGGAAGGCTATCTGTTTGAAATGGAACGTCGTGGTTATCTGCAGGCGGGTAGCTTTGTTCCGGAAGTTGCACTGGATAATCCTGATGTGCTGAAACAGACCTATCGTGATTTTATGCTGGCAGGTTCTGATGTGGTTCTGGCATTTACTTATAATGCCCATCGTGAAAAAATGCGTATTATTGGTAAAGAAGATCTGCTGGAACCGCTGAATCGTAGCGCAATTCGTCTGGCAAAAGAAGTTGCAAAAGAACATCCGGAAGAAGAAGCACTGGTTGCAGGTAATATTAGCAATACCAATATTTTTGATCCGGAAGATCCGGCAAGCAAAGAACAGGTTCGTCAGATGTTTCGTGAAATGGTTCAGTGGAGCAAAGAAGAAGGTGTTGATTTTGTTAATGCAGAAACCTTTTATTATCATGAAGAAGCAGTTATTGCACTGGAAGAAATTCTGCGTCAGGATCTGCCGGCAGTTGTTACCCTGGGTGTTATGGGTGAAAATAAAATGCGTGATGGTCTGAGCCCGGAAGAAAGCTGTCGTCTGCTGAGCGAAAAAGGTGCACTGGTTGTTGGTATGAATTGTTTTCGTGGTCCGGATACCATGCAGCCGTTTCTGGAACGTATTCGTCATCATGTTGATGGTTATGTTGCAGGTCTGCCGGTTCCGTATCGTACCAAAGATGATCATCCGACCTTTTTTAATCTGCCGGATAGCGGTTGTAGCTGTCATCTGCCGACCGAAACCACCTTTCCGACCAGCCTGGATCCGCTGTATCATAATCGTTATGAACTGGCAGAATGGGCAAAAGAAGCAAAAGCAATTGGTATTAATTATATTGGTCTGTGTTGTGGTGCAAGCCCGGCAATGATTCGTGCAGTTGCAGAAGCAACCGGTAAAGAAGCAGTTAATAGCACCTATAGCCCGGATATGACCAAACATTTTCTGTTTGGTAAAGATGATAGCCTGAAAGGTCATAATCAGGATTATCGTAGCAAAGCA**TAA**

**Sequence S6. The codon-optimized sequence of *TnBHMT* (*Thioclava nitratireducens*) with an RBS of Ptac**

GACCACAACGGTTTCCCTCTACAAATAATTTTGTTTAACTTTCGCGCGCGTAACAGGAGGAATTAA**ATG**AACGATAACAAACTGACCCGTCTGCTGGATGCCCGTCCGGTTATTTGCGCCGAAGGTTTTCTGTTTGAACTGGAACGTCGCGGTTATCTGACCGCGGGCGAATTTGTTCCGGAAGTTGCCCTGGAATATCCTCAGGCACTGCGTAATCTGCATGTTGATTTTCAGCGTGCGGGTTCTGATATTGTGGAAGCGTTTACTTATAATGGTCATCGTGAAAAAATGCGTGTTATTGGTAAAGAAGATCTGCTGGAACCGCTGAATCGTGCAGCACTGAAAATTGCACGTGAAGTTGCAGATGCAAAAACCGGTAATCTGATGGCAGGTAATATTAGCAATACCAATATTTGGGATCCGGCAGATCCGGCACGTCAGGCAGAAGTTCGTGCAATGTTTGATGAAATGGTTGGTTGGGCAGTTGAAGAAGGTGCAGATATTCTGATTGGTGAAACCTTTTATTATGCAGGTGAAGCACTGTGTGCACTGGAAGCAGCAAAAGCAAGCGGTCTGCCGGTTGTTCTGACCCTGGCACCGATGGCAGCAAATGAAATGATGGATGGTGTTGGTATTGTTGAAACCTGTCAGAAACTGGAACAGGCAGGTGCAGATGTTGTTGGTCTGAATTGTTTTCGTGGTCCGCAGACCATGATGCCGTGGCTGCGTAAAGTTCGTGCAGCAGTTAGCTGTCATGTTGGTGCACTGCCGGTTCCGTATCGTACCACCAAAGAAGAACCGACCTTTTTTAATCTGAGCGATCATAATGGTTGTACCTGTCCGAGCCCGCATGGTCGTACCTTTCCGACCGCACTGGATCCGCTGGCATGTAATCGTTATGAAATTGGTGCATTTGCACGTGAAGCACAGGCAATTGGTGTTAATTATCTGGGTGTTTGTTGTGGTGCAAGCCCGATGCATATTCGTGAAATGGCAGAAGCAGTTGGTCGTGAAACCGAAGCAAGCCGTTTTAGCGAACGTATGGAAAATCATTTTATGTATGGTCAGAATGATCGTCTGCCGGCACATATTCGTGCACTGGGTGATGGTGCA**TAA**

**Sequence S7. The sequence of promoter Ptac**

CTGGATAACTTGACAATTAATCATCGGCTCGTATAATGTGTGGAGGGAGACCACAACGGTTTCCCTCTACAAATAATTTTGTTTAACTTTCGCGCGCGTAACAGGAGGAATTAA

**Sequence S8. The sequence of the RBS of Ptac**

GACCACAACGGTTTCCCTCTACAAATAATTTTGTTTAACTTTCGCGCGCGTAACAGGAGGAATTAA

**Sequence S9. The sequence of the RBS of Ptrc**

GAGCGGATAACAATTTCACACAGGAAACAGACC

**Sequence S10. The sequence of the RBS of promoter T7**

CTCTAGAAATAATTTTGTTTAACTTTAAGAAGGAGATATACC
